# Hydroxysafflor Yellow A Induces Apoptosis and Inhibits Inflammatory Cytokine Expression in Rheumatoid Arthritis Synovial Fibroblasts Through the MEK‐ERK Pathway

**DOI:** 10.1155/mi/7413982

**Published:** 2026-06-08

**Authors:** Dongwei Li, Hongkai Yang, Yao Feng, Xiaoda Liu, Feng Wang, Yue Jiang, Suqin Wu, Xifan Mei

**Affiliations:** ^1^ Department of Pharmacy, Liaoning Institute of Basic Medical Sciences, Shenyang, 110000, China; ^2^ Department of Pharmacy, Liaoning Vocational College of Medicine, Shenyang, 110000, China; ^3^ Department of Orthopedic, Third Affiliated Hospital of Jinzhou Medical University, Jinzhou, 121001, China, jzmu.edu.cn; ^4^ Laboratory of Pathological Anatomy, Liaoning Institute of Basic Medical Sciences, Shenyang, 110000, China; ^5^ Department of Morphology, Liaoning Vocational College of Medicine, Shenyang, 110000, China; ^6^ Department of Immunology, Liaoning Institute of Basic Medical Sciences, Shenyang, 110000, China; ^7^ Department of Immunology and Microbiology, Liaoning Vocational College of Medicine, Shenyang, 110000, China

## Abstract

**Objective:**

The objective of this study was to investigate whether hydroxysafflor yellow A (HSYA) affects the proliferation and apoptosis of fibroblast‐like synoviocytes (FLSs) in rheumatoid arthritis (RA). Synovial fibroblast hyperplasia is a key pathological feature of RA, and its inhibition may slow disease progression. Although HSYA has attracted significant interest, its effects on RA are not yet fully understood. Thus, we performed a series of experiments to examine the impact of HSYA on FLSs.

**Methods:**

To investigate the effects of HSYA on FLSs, we exposed FLSs to Interleukin‐1 beta (IL‐1β) and assessed their proliferation via CCK8, scratch, transwell, TUNEL, and flow cytometry assays. Inflammatory cytokine levels were determined by ELISA. The involvement of the MEK‐ERK signaling pathway was validated via RT‐qPCR and immunofluorescence analyses.

**Results:**

Treatment with HSYA significantly inhibited FLSs proliferation, while promoting their apoptosis. Moreover, HSYA altered the expression of the inflammatory cytokines IL‐6, IL‐10, and TNF‐α in FLSs. The anti‐inflammatory and anti‐proliferative effects of HSYA on FLSs were mediated, at least in part, through the inhibition of the ERK signaling pathway.

**Conclusion:**

Our findings demonstrate that HSYA induces apoptosis, inhibits proliferation, and suppresses the production of inflammatory cytokines in FLSs via the ERK/MEK signaling pathway. These findings suggest that HSYA has potential therapeutic value in the treatment of RA and warrants further investigation.

## 1. Introduction

Rheumatoid arthritis (RA) is a chronic inflammatory disease that causes severe joint damage and extra‐articular complications. Patients with RA suffer significant physical, psychological, and socioeconomic consequences. The synovium, the membrane lining the inner surface of the joint capsule, is composed of the extracellular matrix, cells, and fibroblast‐like synoviocytes (FLSs) [1, 2]. In RA, the synovium undergoes pathological changes, including hyperplasia and chronic inflammation [3, 4], the most significant of which is FLSs proliferation. FLSs play a critical role in the pathogenesis of RA by synthesizing a range of cytokines and chemokines [5, 6], including NF‐κB, RANKL [1], and Dickkopf‐related protein 1 [7], which promote osteoclastogenesis and inhibit osteoblast activity, ultimately contributing to the joint destruction characteristic of RA.

Hydroxysafflor yellow A (HSYA), a significant water‐soluble component of safflower extracts, has been identified as a potent bioactive compound [8, 9]. Previous studies have revealed that HSYA can modulate several critical inflammatory processes in macrophages, including reverse cholesterol transport, oxidative stress [10], inflammasome activity, autophagy, and fatty acid synthesis [11–13], thereby preventing their activation and release of inflammatory mediators.

The mitogen‐activated protein kinase (MAPK) signaling pathway is a key intracellular signaling network that mediates a variety of cellular functions such as proliferation, differentiation, the cell cycle, survival, and apoptosis [14]. The ERK1 and ERK2 kinases form part of this pathway and are implicated in the pathogenesis of RA [15]. The RAF‐MEK‐ERK cascade, in particular, is essential for regulating these processes and has been implicated in the development of RA [16], as well as in the modulation of FLSs proliferation and inflammatory responses [17, 18].

Our study aimed to elucidate the effects of HSYA on FLSs and its underlying mechanisms of action, which may provide a valuable foundation for future research and clinical applications of HSYA as a potential therapeutic agent for RA.

## 2. Materials and Methods

### 2.1. Cell Culture

FLSs were procured from Shanghai Hongshun Biotechnology Co., Ltd. The cells were cultured in DMEM supplemented with 10% FBS, 100 U/mL penicillin, and 100 μg/mL streptomycin and maintained in a humidified incubator at 37°C and 5% CO_2_ with regular medium changes every 2 days.

### 2.2. Establishment of the Cell Model

To determine the optimal concentration of IL‐1β to induce an inflammatory response in FLSs, the cells were seeded in 96‐well plates and treated with various concentrations of IL‐1β (0, 1.25, 2.5, 5, 10, 20, or 40 μg/L) for 12, 24, or 48 h. To assess cell viability, 10 μL of CCK‐8 solution was added to each well,and the absorbance at 450 nm was measured via a microplate reader after 2 h of incubation. The IL‐1β concentration that induced optimal cell activation was identified to be 5 μg/L on the basis of this experiment (Figure S1 and Table S1).

### 2.3. Cell Proliferation

After FLSs were seeded in 96‐well plates and allowed to reach 70%–80% confluence, the cells were divided into four groups: control (treated with 5 μg/L IL‐1β), low‐dose HSYA (LH; 2 μmol/L HSYA), medium‐dose HSYA (MH; 10 μmol/L HSYA), and high‐dose HSYA (HH; 50 μmol/L HSYA). Following 24 and 48 h of treatment, 100 μL of medium supplemented with 10 μL of CCK‐8 solution was added to each well and incubated for 2 h. The absorbance at 450 nm was then measured for each well via a microplate reader to assess cell viability.

### 2.4. Cell Scratch

Logarithmic‐phase FLSs were seeded into 6‐well plates and cultured in a 37°C incubator until the cells reached 70%–80% confluence. The cells in each well were then subjected to serum‐free starvation for 12 h. A scratch was then created via a 200 μL pipette tip at 0 h, and the cultures were maintained for 24 and 48 h. At each timepoint, images of the scratch wound were taken under the same conditions. Cell migration was quantified by calculating the healing area of each scratch via ImageJ software.

### 2.5. Cell Migration

FLSs in the logarithmic growth phase were treated with different concentrations of HSYA for 24 h. The cell density was then adjusted to 3 × 10^4^ cells/mL, and 200 μL of cells was seeded into the upper chambers of each group. A total of 600 μL of complete medium containing 20% FBS was added to the bottom chambers of each group. After 48 h of migration, the chambers were incubated at room temperature for 15 min, and the number of migrated cells in each group was quantified via a microscope.

### 2.6. Cell Invasion

Matrigel (356234, Corning Company) was diluted in cold DMEM at a ratio of 1:8, and 45 μL of the diluted Matrigel solution was evenly distributed in each chamber to cover the micropores and avoid the formation of air bubbles. The plate was then incubated at 37°C to solidify the Matrigel membrane. Any remaining liquid was carefully aspirated, and 20 μL of serum‐free medium was added to each chamber to hydrate the gel matrix. This medium was then aspirated before being used in the invasion assay. The remaining steps were combined with the migration experiment.

### 2.7. TUNEL Staining

For each sample, 100 μL of the TdT equilibrium buffer was added, and the reaction was carried out at 37°C for 20 min. Afterward, 50 μL of the working solution was added, and the reaction was allowed to proceed in the dark at 37°C for 1 h with evaporation being prevented. Following the reaction, excess liquid was absorbed with absorbent paper, and DAPI working solution was added for 5 min of incubation in the dark at room temperature.

### 2.8. Cell Cycle Detection

FLSs were collected and transferred to 1.5 mL centrifuge tubes, which contained ~50 μL of PBS. The cells were gently dispersed by tapping the bottom of the tubes. One milliliter of precooled 70% ethanol was added, followed by gentle mixing. The cells were then fixed overnight at 4°C. After fixation, 500 μL of PI staining solution was added to each sample, which was subsequently incubated in the dark at 37°C for 30 min. Following filtration, a flow cytometer was used to detect the cell cycle in each group [19].

### 2.9. Determination of IL‐6, IL‐10, and TNF‐α Levels in the Cell Supernatant

After FLSs were stimulated with IL‐1β for 48 h, the supernatants from each group were collected at 3000 rpm/min for 15 min. The supernatants were then centrifuged, and the reagents were processed according to the manufacturer’s instructions. The OD values of each well were then measured at a wavelength of 450 nm. The standard curve was used to calculate the corresponding contents of the samples.

### 2.10. RT‒qPCR

cDNA was synthesized from FLSs samples via the Servicebio RT First‐Strand cDNA Synthesis Kit (Wuhan Servicebio Technology), following the manufacturer’s protocol. RT‒qPCR was performed via the SYBR Green PCR Premix Kit (Wuhan Servicebio Technology) and the CFX RT‒qPCR Detection System (Bio‐Rad Laboratories, Hercules, CA, USA). The recommended cycling conditions provided in the kit instructions were used (Table S2).

### 2.11. Immunofluorescence

FLSs were fixed with 4% paraformaldehyde at room temperature for 20 min. After the removal of the fixative, the cells were washed three times with PBS for 5 min each. Primary antibodies against ERK and phosphorylated ERK (p‐ERK) were added, and the samples were incubated overnight at 4°C. The next day, the cells were washed and incubated with the appropriate secondary antibodies at 37°C in the dark for 1 h. Finally, the cells were stained with DAPI staining solution for 15 min in the dark.

### 2.12. Western Blotting

The BCA protein assay kit (EpyZime, Shanghai, China) was utilized to acquire protein samples from FLS, adhering to the manufacturer’s guidelines and standard procedures. The proteins underwent separation through a 10% SDS‐PAGE gel and were then moved onto a polyvinylidene fluoride membrane (Bio‐Rad). Following a 3 h room temperature blockage using 5% skim milk, the membrane underwent an overnight incubation at 4°C (ImmunoWay Biotechnology Co., Plano, TX, USA) with antibodies aimed at p‐Raf 1, MEK, p‐MEK, ERK, p‐ERK (1:500), and actin (1:1000; EpyZime). The detection of immunoreactive protein bands was achieved through an ultra‐high‐sensitivity ECL kit (GLPBIO, Montclair, CA, USA) and a Tanon 5200 chemiluminescence imaging system (Tanon Science and Technology Co., Shanghai, China). The intensity of the blot was measured through ImageJ software using β‐actin as the internal standard.

### 2.13. Statistical Analysis

Statistical analyses were performed via SPSS 19.0 software (SPSS, Inc., Chicago, IL, USA). The data are presented as the mean ± standard deviation (SD). Independent sample *t*‐test tests were used for comparisons between two groups, and one‐way ANOVA was used for comparisons between more than two groups. A *p*‐value less than 0.05 was considered statistically significant.

## 3. Results

### 3.1. HSYA Inhibits the Proliferation, Migration, and Invasion of FLSs

**Figure 1:**
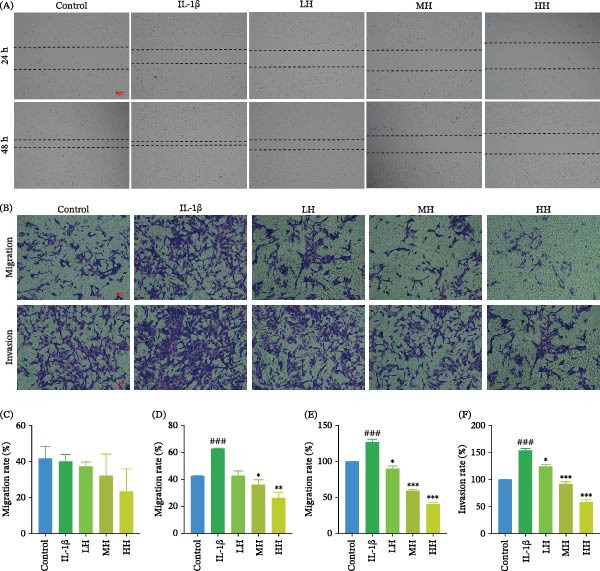
HSYA inhibits the proliferation, migration, and invasion of FLSs. (A) A scratch assay was used to examine the effect of HSYA on FLSs migration. (B) Transwell assays were used to evaluate the effect of HSYA on FLSs migration and invasion. (C) The results of the scratch assay were analyzed after 24 h. (D) The results of the scratch assay were analyzed after 48 h. (E) Analysis of FLSs migration in different groups. (F) Analysis of FLSs invasion in different groups. LH, MH, and HH groups were all tested in IL‐1β–treated FLSs. The data are expressed as the SEM ± mean (*n* = 3). ^###^ *p*  < 0.01 compared with the control group.  ^∗^ *p*  < 0.05,  ^∗∗^ *p*  < 0.01, and  ^∗∗∗^ *p*  < 0.001 compared with IL‐1β‐treated cells.

To examine the effects of HSYA on FLSs proliferation, a CCK‐8 assay was performed. At 12 and 24 h, there were no significant differences in cell proliferation or viability between the HSYA‐treated groups (MH and HH) and the control group. However, after 48 h, the cell proliferation of the MH and HH groups was significantly lower than that of the IL‐1β‐treated group (Figure S2). As shown in Figure 1A, the addition of HSYA at 24 h reduced the IL‐1β‐induced proliferation of FLSs, but the difference was not statistically significant (Figure 1C). After 48 h, the proliferation of FLSs was inhibited in the MH and HH groups. A scratch assay revealed that the scratch area was significantly smaller in the MH and HH groups than in the IL‐1β group (Figure 1D). In a Transwell assay, we verified the inhibitory effect of HSYA on the migration and invasion of FLSs induced by IL‐1β (Figure 1B). Compared with that of the control group, the migration rate of cells treated with IL‐1β was significantly greater. After treatment with HSYA at MH and HH doses, the number of cells that passed through the chamber significantly decreased in a dose‐dependent manner (Figure 1E). As the concentration of HSYA increased, the rate of invasion of cells through the matrix gel membrane decreased, with a significant reduction observed in the MH and HH groups (Figure 1F). Taken together, these results suggest that HSYA has an inhibitory effect on the proliferation, migration, and invasion of FLSs.

### 3.2. HSYA Promotes Apoptosis of FLSs

**Figure 2:**
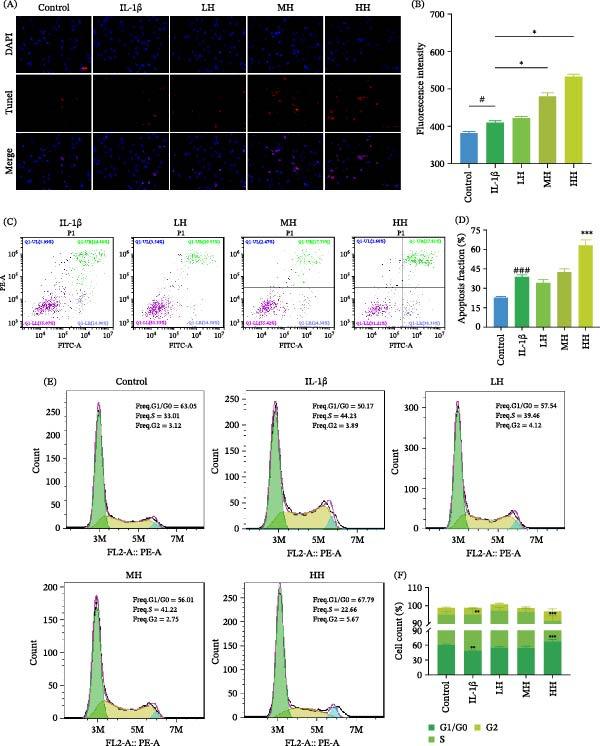
HSYA promotes FLSs apoptosis. (A) A TUNEL assay was used to detect apoptosis in FLSs from each group. (B) Quantitative analysis of TUNEL results showing percentage of apoptotic FLSs in each group. (C) Flow cytometry with Annexin V‐PI double staining was used to detect apoptosis in FLSs from each group, with the apoptotic fraction analyzed in (D). (E) Flow cytometry was used to detect the cell cycle distribution of FLSs in each group, and the results were analyzed in (F). LH, MH, and HH groups were all tested in IL‐1β–treated FLSs. The data are expressed as the SEM ± mean (*n* = 3). ^#^ *p*  < 0.05, ^##^ *p*  < 0.01, and ^###^ *p*  < 0.001 compared with the control conditions,  ^∗^ *p*  < 0.05 and  ^∗∗∗^ *p*  < 0.001 compared with the IL‐1β‐treated cells.

A TUNEL assay was used to assess the effect of HSYA on FLSs apoptosis (Figure 2A). Compared with that of the control group, the rate of IL‐1β‐induced FLSs apoptosis was greater. Additionally, the MH and HH groups presented increased apoptosis rates compared with those of the IL‐1β‐treated group. However, no significant change was observed in the LH group (Figure 2B). These findings were consistent with the results of the Annexin V‐PI double‐staining flow cytometry assay (Figure 2C). Taken together, these results suggest that HSYA induces FLSs apoptosis in a dose‐dependent manner. The rate of FLSs apoptosis increased after treatment with IL‐1β. Compared with the IL‐1β‐treated FLSs, the LH‐ and MH‐treated FLSs did not significantly differ in terms of apoptosis rates, whereas the HH‐treated FLSs exhibited a significantly increased apoptosis rate (Figure 2D). Western blot results further confirmed that HSYA promotes apoptosis of FLSs, at least in part through the activation of caspase‐3 (Figure S3). These findings suggest that HSYA may induce FLSs apoptosis in a dose‐dependent manner.

HSYA promoted FLSs apoptosis, as demonstrated by flow cytometry analysis of the cell cycle distribution (Figure 2E). Compared with that in the control group, the proliferation of FLSs induced by IL‐1β was accelerated, with a significant increase in the number of S‐phase cells. There was no significant difference in the number of FLSs in the G2/M phase between HH and IL‐1β‐induced FLSs, whereas the number of FLSs in the S phase significantly decreased (Figure 2F). These results indicate that IL‐1β increases FLSs proliferation by accelerating the S phase of the cell cycle, whereas having minimal effect on the G2/M phase. In the HH group, the number of cells in the S phase significantly decreased, while the proportion of cells in the G0/G1 phase increased. These findings suggest that HSYA may interfere with the cell cycle progression of IL‐1β‐induced FLSs, which could contribute to the promotion of apoptosis and the inhibition of proliferation.

### 3.3. HSYA Inhibits the Expression of Inflammatory Cytokines in FLSs

**Figure 3:**
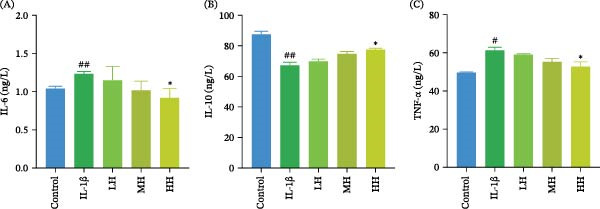
Effects of HSYA on the levels of cytokines induced by IL‐1β in FLSs. (A) Expression of IL‐6 in FLSs. (B) Expression of IL‐10 in FLSs. (C) Expression of TNF‐α in FLSs. LH, MH, and HH groups were all tested in IL‐1β–treated FLSs. The data are expressed as the SEM ± mean (*n* = 3). ^#^ *p*  < 0.05 and ^##^ *p*  < 0.01 compared with the control group.  ^∗^ *p*  < 0.05 compared with IL‐1β‐treated cells.

To investigate the release of inflammatory cytokines in FLSs following stimulation with IL‐1β, ELISAs were performed on the supernatant of FLSs cultures (Figure 3). IL‐6 and TNF‐α, both inflammatory cytokines, were significantly elevated in the IL‐1β‐induced group compared with the control group (Figure 3A,C). These results suggest that IL‐1β is a potent inducer of inflammatory mediators in FLSs, potentially contributing to inflammatory diseases such as RA. In addition to elevated levels of proinflammatory cytokines, the IL‐1β‐induced group also presented significantly reduced levels of the antiinflammatory cytokine IL‐10 (Figure 3B). Compared with those in the IL‐1β‐induced group, the concentrations of IL‐6 and TNF‐α were significantly lower, whereas the level of IL‐10 was significantly greater in the FLSs in the HH group (Figure 3A–C). Collectively, the data from this study suggest that HSYA exerts anti‐inflammatory effects by modulating the production of cytokines in FLSs. The inhibitory effect of HSYA on proinflammatory cytokines, such as IL‐6 and TNF‐α, combined with its stimulatory effect on the anti‐inflammatory cytokine IL‐10, suggest that HSYA may be a promising candidate for the treatment of inflammatory diseases associated with excessive synovial cell proliferation, including RA.

### 3.4. HSYA Impacts on FLSs Through the ERK Pathway

**Figure 4:**
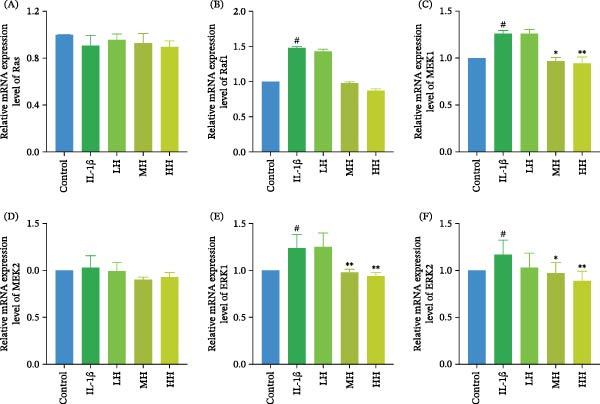
Effects of HSYA on the relative expression of ERK signaling pathway mRNAs in FLSs. (A) Ras, (B) Raf1, (C) MEK1, (D) MEK2, (E) ERK1, and (F) ERK2. LH, MH, and HH groups were all tested in IL‐1β–treated FLSs. The data are expressed as the means ± SEM (*n* = 3). ^#^ *p*  < 0.05 compared with the control group.  ^∗^ *p*  < 0.05 and  ^∗∗^ *p*  < 0.01 compared with the IL‐1β‐treated group.

To uncover the potential underlying molecular mechanisms responsible for the observed effects of HSYA on FLSs, we utilized RT‒qPCR to examine the expression of genes involved in the ERK signaling pathway. Our results revealed that treatment of FLSs with IL‐1β for 48 h resulted in increased mRNA expression of the ERK pathway components Raf1, MEK1, ERK2, and ERK1 relative to that in the control group (Figure 4). Treatment with MH or HH led to a dose‐dependent reduction in the mRNA expression levels of these ERK pathway genes, suggesting that the inhibition of ERK signaling by HSYA may be a contributing factor to its anti‐inflammatory effects.

**Figure 5:**
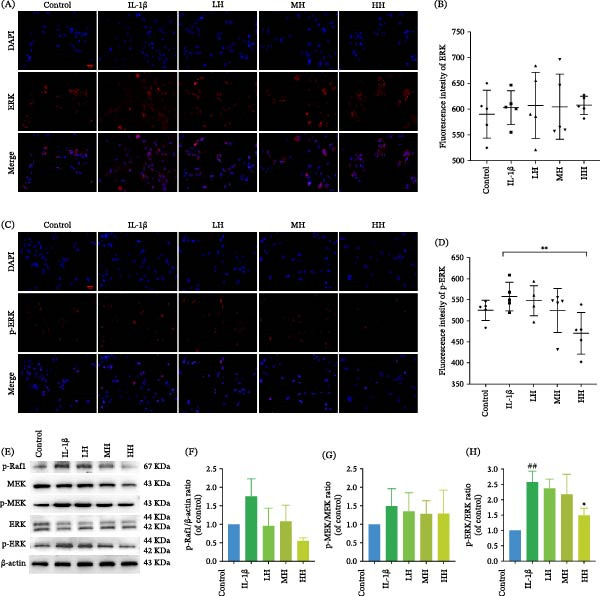
Effects of HSYA on the relative expression of ERK signaling pathway proteins in FLSs. (A) Immunofluorescence assessment of the effect on ERK expression in FLSs from each group. (B) Quantitative analysis of ERK expression via immunofluorescence. (C) Immunofluorescence assessment of the effect on p‐ERK expression in FLSs from each group. (D) Quantitative analysis of p‐ERK expression in immunofluorescence. (E) Effects of HSYA on the ERK signaling pathway protein expression in FLS assessed by Western blotting. (F–H) Quantitative analysis of the expression of p‐Raf1/β‐actin, p‐MEK/MEK and p‐ERK/ERK. LH, MH, and HH groups were all tested in IL‐1β–treated FLSs. Data represent mean ± SEM (*n* = 3). ^##^ *p*  < 0.01 compared with control group,  ^∗^ *p*  < 0.05 and  ^∗∗^ *p*  < 0.01 compared with IL‐1β treated group.

To further confirm the effect of HSYA on the ERK signaling pathway, we conducted an immunofluorescence experiment to investigate the protein expression of ERK1/2 and p‐ERK1/2 in FLSs. Immunofluorescence analysis revealed no significant differences in the protein expression of ERK1/2 in the nuclei or cytoplasm of cells among the different treatment groups (Figure 5A,B). However, IL‐1β stimulation resulted in increased expression of the p‐ERK1/2 protein in the nuclei of FLSs (Figure 5C,D), which was significantly reduced by treatment with HH concentrations of HSYA. These results provide additional evidence to support the mechanism of action of HSYA in its anti‐inflammatory effects on FLSs.

The results of western blotting (Figure 5E) showed that there was no difference between the control group and the IL‐1β group, and the expression of p‐Raf1 was significantly decreased between the HH and the IL‐1β treatment group (Figure 5F). There was no significant difference in p‐MEK/MEK activity between the groups (Figure 5G). Compared with the control group, the p‐ERK/ERK in the IL‐1β group increased significantly by 57% (Figure 5H), and the degree of down‐regulation of p‐ERK/ERK in each HSYA group was dose‐dependent, and the HH was the most significant. The expressions of ERK and p‐ERK were consistent with the results of immunofluorescence. These results indicated that HSYA could down‐regulate the expression of p‐MEK/MEK and p‐ERK/ERK proteins, thereby inhibiting the ERK signaling pathway.

## 4. Discussion

RA can affect people of all ages, but the exact cause remains unknown [20]. However, research has shown that the proliferation and migration of FLSs play key roles in the pathogenesis of RA [21, 22]. As traditional Chinese medicine (TCM) becomes increasingly popular, more researchers are investigating the potential of TCM components as therapies for RA. While HSYA is the primary active component of safflower, it has been largely unexplored in the context of RA research, with Li et al. [23] being a notable exception. HSYA has demonstrated anti‐inflammatory properties and potential therapeutic effects in RA. However, the exact molecular mechanisms underlying its anti‐RA activity remain incompletely defined.

In our study, we discovered that HSYA exerts multiple effects on FLSs. Specifically, HSYA inhibited proliferation and migration, promoted apoptosis, and suppressed the expression of inflammatory factors in FLSs [24]. Maintaining the inflammatory phenotype of FLSs in vitro is crucial for modeling RA. Research has demonstrated that IL‐1β promotes synovial cell proliferation and differentiation [25], leading to the production of prostaglandin E2 (PGE2) and collagenase, which cause cartilage degradation and synovial inflammation. We used IL‐1β stimulation to mimic the RA inflammatory environment, enabling us to investigate HSYA under pathologically relevant conditions.

One prominent factor in RA progression is the abnormal resistance of FLSs to apoptosis, which contributes to excessive proliferation and pannus formation [26]. Normally, quiescent cells reside in the G0/G1 phase, whereas cells in the S and G2/M phases undergo DNA replication and division, respectively. Disruption of this balance—particularly accelerated entry into the S phase—can promote pathological synovial hyperplasia. Blocking the cell cycle in the S phase has been reported to trigger apoptosis and suppress proliferation [27]. In our study, IL‐1β stimulation markedly increased the proportion of FLSs in the S phase, indicating enhanced proliferative activity. Treatment with HSYA reversed this effect by significantly reducing the proportion of S‐phase cells and concomitantly increasing the proportion of cells in the G0/G1 phase, while no notable changes were observed in the G2/M phase. These findings suggest that HSYA interferes with the cell cycle progression of IL‐1β‐induced FLSs, thereby attenuating their abnormal proliferation and facilitating apoptosis. However, because our study did not include the mechanistic claim regarding cell cycle blockade, it remains preliminary and requires further validation.

At the signaling level, numerous studies have established the central role of the MAPK pathway—particularly ERK—in RA pathogenesis as it regulates proliferation, apoptosis, and cytokine release in synovial cells [28]. In our study, HSYA inhibited ERK phosphorylation, suggesting that its effects may be mediated through the suppression of ERK/MAPK signaling. Downstream of ERK, transcription factors such as AP‐1 and NF‐κB are known to regulate the expression of inflammatory cytokines and cell survival genes [29]. Thus, HSYA’s inhibition of ERK may reduce proinflammatory cytokines (IL‐6 and TNF‐α) and enhance anti‐inflammatory cytokines (IL‐10), which is consistent with our findings.

Compared with current RA therapies, HSYA’s mechanism appears to be distinct but complementary. Biologics such as TNF inhibitors (e.g., etanercept and adalimumab) and IL‐6 receptor blockers (e.g., tocilizumab) act by neutralizing specific cytokines, while JAK inhibitors (e.g., tofacitinib) suppress broad cytokine signaling [30]. In contrast, HSYA targets intracellular MAPK signaling, suggesting a different point of intervention that could complement existing strategies. Nevertheless, unlike approved therapies, our data are limited to in vitro models.

The limitations of this study should be acknowledged. Only ERK/MAPK signaling was assessed, while other MAPK branches such as p38 and JNK, both of which are implicated in RA pathogenesis, were not examined [31]. All experiments were performed on immortalized synovial cell lines rather than patient‐derived primary FLSs, and no in vivo validation was performed. These factors restrict the translational relevance of our findings.

Future research should therefore include validation of apoptosis‐related proteins (caspases, Bcl‐2 family, and cyclins/CDKs) to clarify HSYA’s role in cell cycle regulation; exploration of additional MAPK branches (p38 and JNK) and cross‐talk with NF‐κB signaling; and studies using primary RA‐FLSs and animal models to better approximate clinical settings. Ultimately, integrating HSYA into the broader therapeutic landscape will require systematic mechanistic dissection and translational evaluation.

## 5. Conclusion

In conclusion, our research suggests that HSYA can inhibit the proliferation of FLSs and promote their apoptosis through the regulation of the ERK/MAPK signaling pathway. These findings provide a novel theoretical basis for the potential treatment of RA with HSYA.

## Funding

This study was funded by the Youth Project of 2021 Scientific Research of the Education Department of Liaoning Province (No. LJKQZ2021163).

## Conflicts of Interest

The authors declare no conflict of interest.

## Supporting Information

Additional supporting information can be found online in the Supporting Information section.

## Supporting information


**Supporting Information** Figure S1: Proliferation of RA‐FLS cells induced by IL‐1β at different concentrations and time points. Figure S2: Effects of HSYA on RA‐FLS cells proliferation assessed by CCK‐8 assay. Figure S3: HSYA‐induced apoptosis in RA‐FLS cells via caspase‐3 activation. Table S1: Quantitative analysis of RA‐FLS cell proliferation induced by IL‐1β under different conditions. Table S2: Primer sequences used in this study.

## Data Availability

The data that support the findings of this study are available on request from the corresponding author. The data are not publicly available due to privacy or ethical restrictions.
