# Supplementary material for: Hydroxysafflor Yellow A Induces Apoptosis and Inhibits Inflammatory Cytokine Expression in Rheumatoid Arthritis Synovial Fibroblasts Through the MEK‐ERK Pathway
Source: Mediators Inflamm. 2026 Jun 8;2026:7413982. doi: 10.1155/mi/7413982 (PMC13244135; doi:10.1155/mi/7413982)
Supplement: Supplementary file 1 — Supporting Information Figure S1: Proliferation of RA‐FLS cells induced by IL‐1β at different concentrations and time points. Figure S2: Effects of HSYA on RA‐FLS cells proliferation assessed by CCK‐8 assay. Figure S3: HSYA‐induced apoptosis in RA‐FLS cells via caspase‐3 activation. Table S1: Quantitative analysis of RA‐FLS cell proliferation induced by IL‐1β under different conditions. Table S2: Primer sequences used in this study. [file MI-2026-7413982-s001.docx]

**Supplementary material**

**Hydroxysafflor Yellow A Induces Apoptosis and Inhibits Inflammatory Cytokine Expression in Rheumatoid Arthritis Synovial Fibroblasts through the MEK-ERK Pathway**

Dongwei Li^a, b, #^, Hongkai Yang^c, #^, Yao Feng^d, e^, Xiaoda Liu^f, g^, Feng Wang^a, b^, Yue Jiang^f, g^, Suqin Wu^f, g, *^, Xifan Mei ^c, f, g,*^

a: Department of pharmacy, Liaoning Institute of Basic Medical Sciences, Shenyang 110000, China

b: Department of pharmacy, Liaoning Vocational College of Medicine, Shenyang 110000, China

c: Department of Orthopedic, Third Affiliated Hospital of Jinzhou Medical University, Jinzhou 121001, China

d: Laboratory of Pathological Anatomy, Liaoning Institute of Basic Medical Sciences, Shenyang 110000, China

e: Department of Morphology, Liaoning Vocational College of Medicine, Shenyang 110000, China

f: Department of Immunology, Liaoning Institute of Basic Medical Sciences, Shenyang 110000, China

g: Department of Immunology and Microbiology, Liaoning Vocational College of Medicine, Shenyang 110000, China

# Contributed equally

* Corresponding author

**Supplementary Figure1**

The cell proliferation activity (%) of RA-FLS cells induced by IL-1β in different concentrations and time points (*$\bar{\text{x}}$±s, n*=3, %).


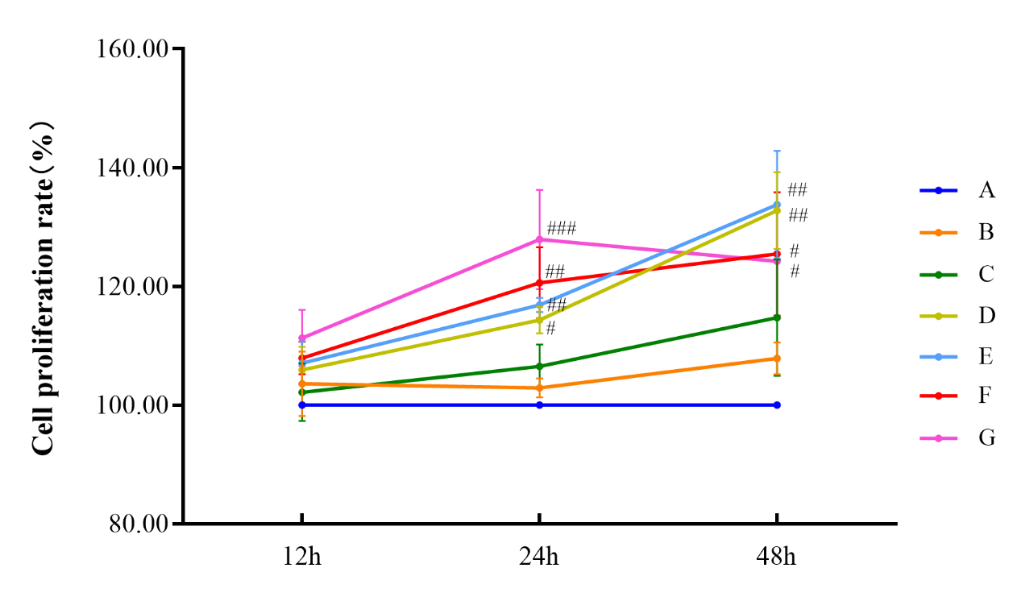


A: 0 μg·L^-1^, B: 1.25 μg·L^-1^, C: 2.5 μg·L^-1^, D: 5μg·L^-1^, E: 10 μg·L^-1^, F: 20 μg·L^-1^, G: 40 μg·L^-1^. ^#^*P*<0.05, ^##^*P*<0.01 and ^###^*P*<0.01 compared with the control condition.

**Supplementary Figure2**

Effect of HSYA on the proliferation of FLS measured by CCK-8 assay.


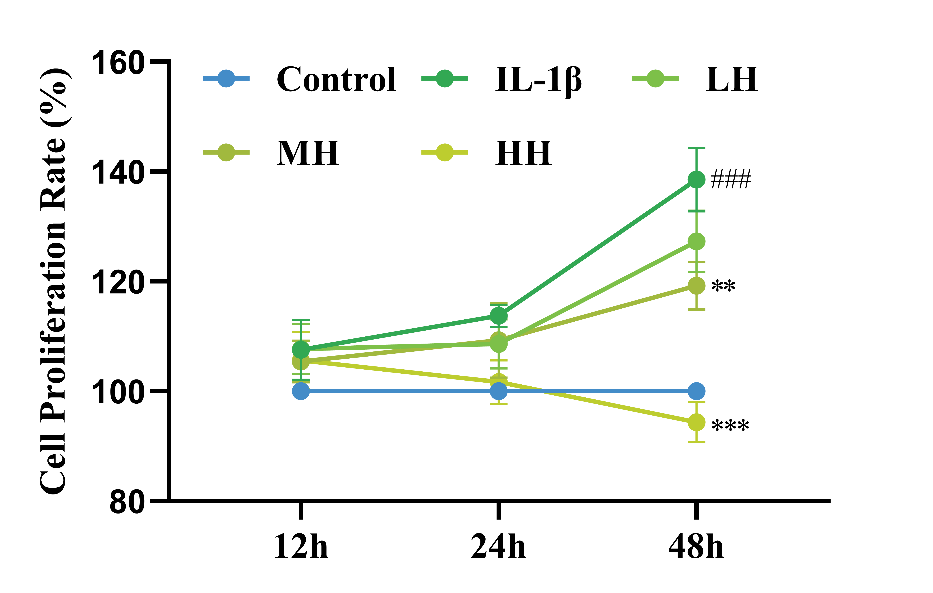


*^###^P<0.01*compared with the control condition. ***P<0.01 and ***P＜0.001* compared with IL-1β treated cells.

**Supplementary Figure3**

HSYA promotes apoptosis through caspase-3 activation.


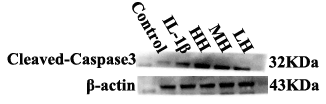


**Supplementary Table 1**

The cell proliferation activity of RA-FLS cells induced by IL-1β in different

concentrations and time points (*$\bar{\text{x}}$±s, n*=3,%)

| IL-1β (μg·L^-1^) | Cell proliferation activity (%) | | |
| --- | --- | --- | --- |
|  | 24 h | 48 h | 72 h |
| A （ 0 ） | 100.00±0.00 | 100.00±0.00 | 100.00±0.00 |
| B （1.25） | 103.59±5.41 | 102.90±1.59 | 107.86±2.69 |
| C （2.5） | 102.13±4.83 | 106.51±3.69 | 114.71±9.83 |
| D （5） | 105.94±3.90 | 114.33±2.25^#^ | 132.79±6.49^##^ |
| E （10） | 107.09±3.65 | 116.86±1.21^##^ | 133.76±9.04^##^ |
| F （20） | 107.94±2.77 | 120.60±6.00^##^ | 125.48±10.42^#^ |
| G （40） | 111.29±4.74 | 127.93±8.39^###^ | 124.23±9.53^#^ |

**Supplementary Table 2**

Primer sequence list

| Gene  name | Primer information | Primer sequence（5’-3’） | Primer length  （bp） |
| --- | --- | --- | --- |
| GAPDH | NM_002046 | Forward：GGAAGCTTGTCATCAATGGAAATC | 168 |
|  |  | Reverse：TGATGACCCTTTTGGCTCCC |  |
| KRAS | NM_001369786.1 | Forward：ATGAGGGACCAGTACATGAGGAC | 208 |
|  |  | Reverse：TTCTTGCTAAGTCCTGAGCCTGT |  |
| Raf1 | NM_001354689.3 | Forward：GGTCAATGTGCGAAATGGAA | 161 |
|  |  | Reverse：CAATCAAAGACGCAGCATCAGTA |  |
| MEK1 | NM_002755.4 | Forward：TGGGAGAACTGAAGGATGACGA | 228 |
|  |  | Reverse：CGCTGTAGAACGCACCATAGAA |  |
| MEK2 | NM_030662.3 | Forward：AGCGGTCACGGGATGGATA | 256 |
|  |  | Reverse：GGTTCAGCCGCAGGGTTTT |  |
| ERK1 | NM_001040056.3 | Forward：GAGAGATGTCTACATTGTGCAGGAC | 202 |
|  |  | Reverse：AATCTTAAGGTCGCAGGTGGTG |  |
| ERK2 | NM_002745.5 | Forward：CCAGACCTACTGCCAGAGAACC | 221 |
|  |  | Reverse：TGAGGATCTGGTAGAGAAAATAGCAG |  |
